# Association between temporomandibular disorders, chronic diseases, and ophthalmologic and otolaryngologic disorders in Korean adults: A cross-sectional study

**DOI:** 10.1371/journal.pone.0191336

**Published:** 2018-01-31

**Authors:** Hyun-Seop Song, Joon-Shik Shin, Jinho Lee, Yoon Jae Lee, Me-riong Kim, Jae-Heung Cho, Koh-Woon Kim, Yeoncheol Park, Hyun Jin Song, Sun-Young Park, Seoyoun Kim, Mia Kim, In-Hyuk Ha

**Affiliations:** 1 Jaseng Spine and Joint Research Institute, Jaseng Medical Foundation, Seoul, Republic of Korea; 2 Department of Korean Rehabilitation Medicine, College of Korean Medicine, Kyung Hee University, Seoul, Republic of Korea; 3 Department of Acupuncture & Moxibustion, Kyung Hee University Hospital at Gangdong, Seoul, Republic of Korea; 4 College of Pharmacy, University of Florida, Gainesville, Florida, United States of America; 5 VIAplus, Siheung, Republic of Korea; 6 Korea University Graduate School of Public Health, Seoul, Republic of Korea; 7 Department of Cardiovascular and Neurological Diseases (Stroke Center), College of Korean Medicine, Kyung Hee University, Seoul, Republic of Korea; Taipei Veterans General Hospital, TAIWAN

## Abstract

**Introduction:**

Temporomandibular disorders (TMDs) are common musculoskeletal conditions in the maxillofacial area. Although strong relationships between TMDs and other pain and diseases exist, few studies have comprehensively assessed the association between chronic diseases, ophthalmologic and otolaryngologic disorders and TMD.

**Methods:**

Of 25,534 individuals included in the fifth Korea National Health and Nutrition Examination Survey (2010–2012), 17,575 aged ≥20 years who completed survey items on TMD symptoms were included for cross-sectional analysis. Logistic regression analysis was performed to assess the association between chronic diseases, ophthalmologic and otolaryngologic disorders and examination findings, and TMD symptoms after adjusting for various confounding variables.

**Results:**

Out of 17,575 participants, 2,059 (11.75%) reported experience of ≥1 TMD symptom(s). Compared to individuals without chronic disease, those with asthma (odds ratio (OR) 1.46; 95% confidence interval (CI) 1.09–1.96), migraine (1.44; 1.26–1.65), osteoarthritis (1.51; 1.20–1.89), thyroid dysfunction (1.49; 1.13–1.96), and depressive symptoms (1.51; 1.29–1.77) had higher ORs for TMD prevalence. Participants with tinnitus (1.97; 1.70–2.27), hearing difficulties (1.55; 1.29–1.87), dizziness (1.52; 1.27–1.82), rhinitis (1.46; 1.28–1.65), and xerophthalmia (1.82; 1.57–2.12) also displayed higher ORs for TMD prevalence. Patients diagnosed with chronic rhinosinusitis upon otolaryngologic examination exhibited an OR of 1.44 (95% CI 1.11–1.87) for TMD prevalence, while that for individuals with abnormal laryngoscopic results was 0.57 (95% CI 0.36–0.90).

**Conclusions:**

These findings imply that TMDs, chronic diseases, and ophthalmologic and otolaryngologic disorders hold various correlations, suggesting the need for multitarget approaches to effectively address this phenomenon.

## Introduction

Temporomandibular disorders (TMDs) are significant problems, not only individually for the patient suffering from the condition, but also collectively for society as they incur high economic costs due to treatment and productivity losses [1]. They cover various pathologies of the temporomandibular joint and its surrounding structures [2]. Diagnosis of TMDs is derived from symptom assessment, and the most frequently used diagnostic classification systems are the Research Diagnostic Criteria for Temporomandibular Disorders (RDC/TMD) and the classification of the American Academy of Orofacial Pain (AAOP) [3]. Recently, the expanded taxonomy classifies disorders as temporomandibular joint, masticatory muscle, headache disorders, and disorders involving associated structures, and was developed through consensus of multiple dental and medical experts [4]. Meanwhile, though the number of patients with mastication difficulty, trismus, head and/or neck pain, and clicking sounds has been increasing, reported prevalence estimates tend to vary depending on the target population and diagnosis system. In the general population, TMDs reportedly occur in one out of two individuals, half of whom are aware of their symptoms. However, only 5% require treatment for symptoms severe enough to disrupt daily life [5]. According to the U.S. National Institute of Dental and Craniofacial Research (NIDCR), 5–12% of the U.S. population currently suffers from or has a history of TMD-related problems [6]. TMDs are also relatively common disorders in Korea, where prevalence estimates of chronic symptomatic TMDs persisting for ≥3 months have been put at 3.1% [7].

As a consequence of its multifactorial etiology encompassing various musculoskeletal and psychosocial factors, more research is being concentrated on the potential association between TMDs and other diseases. The Orofacial Pain Prospective Evaluation and Risk Assessment (OPPERA) study concluded that individuals with a history of low back pain at baseline showed a 50% greater incidence of developing TMDs compared to those without back pain history, while a history of genital pain was associated with a 75% higher TMD incidence. Meanwhile, irritable bowel syndrome was found to predict first-onset TMDs following adjustment for demographic characteristics and pain disorders [8]. As temporomandibular joint pain frequently coexists with pain in one or more other areas, especially migraine/headache, neck pain, low back pain, and joint pain [9], it seems likely that temporomandibular pain develops in conjunction with pain in other parts of the body [10]. In addition to pain, TMDs are regarded to be contributing factors in neuropsychiatric disorders such as anxiety and depression [11]. Though not a typical otolaryngologic condition, TMDs have also been implicated in development of otolaryngological symptoms [12]. A 2008 study in a Japanese sample discovered that allergy may be a risk factor for temporomandibular osteoarthritis/osteoarthrosis [13], suggesting an association with immunologic factors as well.

Numerous study findings point towards a relationship between TMDs and various musculoskeletal disorders and systemic diseases, and treatment of these comorbidities further led to improvement of TMD symptoms. Sherman et al. found that TMD treatment resulted in greater improvement when combined with psychological treatment compared to conventional dental care alone, implying that psychological factors should be factored into TMD treatment [14]. Similarly, Aggarwal et al. reported that a comprehensive treatment plan incorporating cognitive behavior therapy (CBT), posture regulation, and biofeedback components provided greater long-term pain relief to subjects with orofacial pain than usual care [15]. Together, these findings indicate that diagnosis and treatment of TMDs require a multifaceted, interdisciplinary approach.

Previous studies have shown that TMDs are related to such musculoskeletal conditions as joint pain and generalized pain, and neuropsychiatric, otolaryngological, and systemic disorders (e.g. allergy). However, the main body of research tends to focus on assessing its direct relationship with 1 or 2 musculoskeletal pain regions or clinical entities manifesting in immediately surrounding or associated structures. Therefore, the aim of the present study was to cover a wider range of potential relationships between TMDs and chronic diseases, ophthalmologic and otolaryngologic symptoms, and otolaryngologic examination results to gain further insight into the syndrome and obtain a more comprehensive knowledge base to the aim of effective diagnosis and treatment.

## Methods

### Study population and sampling

This dataset used in this study was obtained from the 5th Korea National Health and Nutritional Examination Survey (KNHANES V; 2010–2012) which is conducted yearly by the Korean Ministry of Health and Welfare in a nationally representative sample of South Koreans. The survey is comprised of 3 sections (health survey, nutrition survey, and health examination), and employs a rolling sampling design using a complex, stratified, multistage, probability-cluster survey. Additional information is available in ‘‘The 5th KNHANES (2010–2012) Sample Design” and the 1st-4th Sample Design reports. Data are available on request from the KNHANES website [16].

Of a total 31,596 individuals eligible for participation in KNHANES V, 25,534 (80.8% of the total target population) responded. Of these participants, 17,575 aged ≥20 years who completed survey items on temporomandibular joint symptoms were included in the present cross-sectional analysis.

### TMDs

TMD patients were defined as survey participants who replied that they had experience of temporomandibular joint symptoms over the previous year as indicated by the presence of 1 or more of the following symptoms: (a) clicking sounds in the auricular area during the past year, (b) pressure or pain in the auricular area during the past year, or (c) discomfort opening the mouth during the past year.

### Chronic diseases

Diabetes mellitus, asthma, hypertension, migraine, dyslipidemia, rheumatoid arthritis, osteoarthritis, pulmonary tuberculosis, thyroid dysfunction, depressive symptoms, and atopic dermatitis were assessed for chronic disease prevalence. Diabetes mellitus, asthma, dyslipidemia, rheumatoid arthritis, osteoarthritis, pulmonary tuberculosis, thyroid dysfunction, depressive symptoms, atopic dermatitis, and migraine were considered to be present when the patient indicated a history of the corresponding condition in the survey (lifetime prevalence). Hypertension was defined as systolic blood pressure ≥140 mmHg or diastolic blood pressure ≥90 mmHg as recorded by a nurse from the specialized survey performance team of the Korea Centers for Disease Control and Prevention, or hypertension medication intake.

### Ophthalmologic and otolaryngologic symptoms

The survey included items on ophthalmologic and otolaryngological symptoms such as tinnitus, hearing difficulty, dizziness/balance disorder, rhinitis symptoms, and xerophthalmia (dry eye). Tinnitus, dizziness/balance disorder, rhinitis symptoms, and xerophthalmia were regarded to be present when the patient indicated a history of the condition in the health survey (lifetime prevalence). With regard to hearing difficulty, individuals who selected “some discomfort,” “much discomfort,” or “cannot hear at all” out of the 4 available categories (no discomfort; some discomfort; much discomfort; and cannot hear at all) were considered to have hearing difficulty.

### Otolaryngological and dental examination

In otolaryngological and dental examinations, unilateral hearing loss, pre-nasal astringent pale edematous mucosa, pre-nasal astringent watery rhinorrhea, pre-nasal astringent mucoid or mucopurulent discharge, laryngoscopy findings (normal/abnormal), chronic rhinosinusitis, nasal septum deviation, permanent teeth caries, and periodontal disease were assessed.

Unilateral hearing loss was determined to be positive if the lower average value of pure tone audiometry was ≥40 dB in either ear upon examination by an otolaryngology resident using an automated hearing device. Pre-nasal astringent pale edematous mucosa, pre-nasal astringent watery rhinorrhea, and pre-nasal astringent mucoid or mucopurulent discharge were assessed through the discharge and mucosal condition within the nasal cavity by an otolaryngology resident via endoscopy without applying astringents. Laryngoscopy results were based on examination by an otolaryngology resident via a 4mm 70° endoscope. Chronic rhinosinusitis was defined as when either (a) rhinorrhea or postnasal discharge for ≥3 months, or (b) nasal congestion for ≥3 months were recorded in the rhinology section of the survey; with ≥2 symptoms out of rhinorrhea or postnasal discharge, nasal congestion, facial pain or pressure, and dysosmia, or when an otolaryngology resident indicated presence of nasal polyp via endoscopy after applying astringents. Deviation of the nasal septum was considered positive when an otolaryngology resident indicated asymmetry of the left and right nasal cavity via endoscopic examination following astringent spraying. Caries of permanent teeth were diagnosed through confirmation of ≥1 permanent teeth caries upon dental examination by a public health dentist. Periodontal diseases were likewise diagnosed by a public health dentist through oral examination. Occlusion-malocclusion was not included in the dental examination.

### Covariates

The socioeconomic and lifestyle characteristics assessed in participants enrolled in the survey included age, sex, personal income, education level, occupation, marital status, residential area, smoking status, alcohol consumption, body mass index (BMI), and physical activity. Personal income was classified into quartiles (low, mid-low, mid-high, and high), and education levels were categorized into middle school graduation or lower, high school graduation, and university graduation or higher. Occupation was categorized into manager, specialist or specialty-related worker, office worker, service or sales worker, skilled agricultural or fishery worker, technician or equipment and machine operator, simple laborer, and unemployed (e.g. housewife, student). Marital status was dichotomized into single and married, and residence into dong (city) and eup/myeon (town). With regard to smoking status, individuals who had smoked ≥5 packs of cigarettes in their entire lifetime and continued to smoke were categorized as current smokers, individuals who had smoked ≥5 packs in their lifetime but did not currently smoke as past smokers, and individuals who had never smoked or had smoked <5 packs in their lifetime as non-smokers. Alcohol consumption was binary with individuals who drank ≥1 glass of alcohol/month for the past year classified as drinkers. BMI (kg/m^2^) was assessed as a continuous variable through body measurement, and individuals with a BMI of <18.5, 18.5≤ BMI <25, and BMI ≥25 were categorized as underweight, normal weight, and obese, respectively. Individuals who engage in slightly strenuous or moderate physical activities that involve slightly heavy breathing (e.g. leisurely swimming, doubles tennis, volleyball) over the past week for ≥30 min per session were recognized as regular exercise performers.

### Statistical methods

KNHANES is a nationwide sample survey that applies stratified cluster extraction and sampling weights, and accordingly employed a complex sample data analysis method that utilizes stratified variables, cluster variables, and weight variables as design elements. All data analyses were performed using statistical package SAS version 9.4 (SAS Institute Inc., Cary, NC, USA), and p<0.05 was considered to be statistically significant. Continuous variables are presented as mean and standard error, and nominal variables as frequency. Difference in participant characteristics was assessed using Rao-Scott chi-square test and two-sample t-test where appropriate, and complex sample design logistic regression analysis was performed after adjusting for covariates. Each independent variable was adjusted for by age, sex, personal income, education level, occupation, residence, marital status, BMI, alcohol consumption, smoking status, and physical activity in full models. Age and BMI were factored in as continuous variables, while the other covariates were included as nominal variables in calculation of odds ratios (ORs) and 95% confidence intervals (CIs). The surveyors were not provided with information on surveyees prior to conducting the health survey, and a designated statistician conducted adjustment of analyses with predetermined covariates as selected by clinical relevance by the researchers.

### Ethics statement

All participants gave written informed consent for survey participation. The study protocol conformed to the principles expressed in the Declaration of Helsinki, and was approved by the Institutional Review Board of Jaseng Hospital of Korean Medicine in Seoul, Korea (IRB approval number: KNJSIRB2016-12-014).

## Results

### Population characteristics

Of 17,575 patients who answered the survey, 2,059 (11.75%) had experienced TMD symptoms over the previous year. TMD prevalence differed significantly by age, sex, education level, occupation, marital status, smoking and drinking status, and BMI (Table 1).

**Table 1:** Characteristics of Koreans aged ≥ 20 years participating in KNHANES V (2010–2012) (N = 17,575).

Factors / subgroup		Yes (2,059)		No (15,516)		p value
		N	%	N	%	
Age (years) (mean±SE)		38.68±0.42		47.03±0.25		< .0001*
Sex						
	Male	750	36.43	6,701	43.19	0.0081
	Female	1,309	63.57	8,815	56.81	
Personal income^a^						
	Low	479	23.26	3,723	23.99	0.2617
	Mid-low	546	26.52	3,834	24.71	
	Mid-high	478	23.22	3,921	25.27	
	High	529	25.69	3,854	24.84	
Education level^a^						
	≤Middle school graduation	483	23.46	5,880	37.90	< .0001
	High school graduation	758	36.81	4,855	31.29	
	≥University graduation	806	39.15	4,444	28.64	
Occupation^a^						
	Manager, specialist or specialty-related worker	345	16.76	1,844	11.88	< .0001
	Office worker	235	11.41	1,158	7.46	
	Service or sales worker	235	11.41	1,836	11.83	
	Skilled agricultural or fishery worker	99	4.81	1,327	8.55	
	Technician or equipment and machine operator	142	6.90	1,446	9.32	
	Simple laborer	147	7.14	1,320	8.51	
	Unemployed (e.g. housewife, student)	843	40.94	6,213	40.04	
Marital status						
	Single	1,526	74.11	13,798	88.93	< .0001
	Married	532	25.84	1,706	11.00	
Area of residence						
	Dong (city)	1,694	82.27	12,184	78.53	0.0317
	Eup/myeon (town)	365	17.73	3,332	21.47	
Smoking state^a^						
	Non-smoker	1,336	64.89	9,272	59.76	0.0002
	Past smoker	276	13.40	2,876	18.54	
	Current smoker	439	21.32	3,040	19.59	
Alcohol intake						
	Non-drinker	923	44.83	7,335	47.27	0.0181
	Drinker	1,121	54.44	7,790	50.21	
Body mass index (BMI) (mean±SE)		23.25±0.12		23.74±0.04		< .0001 *
	<18.5	145	7.04	648	4.18	< .0001
	18.5≤BMI<25	1,338	64.98	9,797	63.14	
	≥25	570	27.68	5,009	32.28	
Regular moderate exercise						
	No	1,902	92.37	13,856	89.30	0.0391
	Yes	148	7.19	1,317	8.49	

Continuous variables are given as mean and standard error, and nominal variables as frequency.

p values were calculated using Rao-scott chi-square test, and the level of significance was set at <0.05.

* p value was analyzed by two-sample t-test.

^a^ Missing values for personal income were n = 211 (10.25%); missing values for education n = 349 (16.95%); missing values for occupation n = 385 (18.70%); and missing values for smoking n = 336 (16.32%).

KNHANES V, 5th Korea National Health and Nutritional Examination Survey

### Association between TMD prevalence and chronic diseases

Table 2 presents the relationship between TMD prevalence and chronic diseases. The OR for TMD prevalence was 1.46 in individuals with asthma (95% CI, 1.09–1.96), 1.44 in migraine (95% CI, 1.26–1.65), 1.51 in osteoarthritis (95% CI, 1.20–1.89), 1.49 in thyroid dysfunction (95% CI, 1.13–1.96), and 1.51 in depressive symptoms (95% CI, 1.29–1.77).

**Table 2:** Association between chronic diseases and TMDs in Koreans aged ≥ 20 years participating in KNHANES V (2010–2012).

Chronic disease			Univariate			Full model		
	Subgroup	N (TMD cases)	OR	95% CI		OR	95% CI	
Diabetes mellitus	No (Ref.)	15,744 (1,942)	1			1		
	Yes	1,511 (106)	0.42	0.32	0.56	0.74	0.55	1.00
Asthma	No (Ref.)	16,486 (1,945)	1			1		
	Yes	769 (103)	1.20	0.91	1.59	1.46	1.09	1.96
Hypertension	No (Ref.)	7,242 (1,110)	1			1		
	Yes	9,952 (924)	0.59	0.52	0.67	0.95	0.81	1.11
Migraine	No (Ref.)	13,319 (1,399)	1			1		
	Yes	3,940 (611)	1.57	1.38	1.79	1.44	1.26	1.65
Dyslipidemia	No (Ref.)	15,259 (1,877)	1			1		
	Yes	1,996 (171)	0.56	0.45	0.70	0.94	0.75	1.20
Rheumatoid arthritis	No (Ref.)	16,808 (2,000)	1			1		
	Yes	445 (48)	0.68	0.45	1.01	1.09	0.72	1.66
Osteoarthritis	No (Ref.)	14,689 (1,795)	1			1		
	Yes	2,565 (253)	0.74	0.62	0.88	1.51	1.20	1.89
Pulmonary tuberculosis	No (Ref.)	16,268 (1,939)	1			1		
	Yes	987 (109)	0.73	0.56	0.95	1.00	0.75	1.33
Thyroid dysfunction	No (Ref.)	16,539 (1,949)	1			1		
	Yes	712 (99)	1.19	0.91	1.55	1.49	1.13	1.96
Depressive symptoms	No (Ref.)	14,628 (1,662)	1			1		
	Yes	2,624 (386)	1.32	1.14	1.54	1.51	1.29	1.77
Atopic dermatitis	No (Ref.)	16,491 (1,906)	1			1		
	Yes	761 (142)	1.61	1.28	2.02	1.13	0.89	1.44

Full models were adjusted for age, sex, personal income, education level, occupation, residence, marital status, BMI, alcohol intake, smoking status, and physical activity in estimation of odds ratios (ORs) and 95% confidence intervals (CIs).

Frequencies were analyzed for each variable using frequency analysis, and ORs and 95%Cis were calculated by complex sample design.

TMD, temporomandibular disorder; KNHANES V, 5th Korea National Health and Nutritional Examination Survey; OR, odds ratio; CI, confidence interval

### Association between TMD prevalence and ophthalmologic and otolaryngological symptoms

Table 3 shows the relationship between TMD prevalence and eye, ear, nose, and throat-related symptoms. The OR for TMD prevalence was 1.97 in cases with tinnitus (95% CI, 1.70–2.27), 1.55 in hearing difficulty (95% CI, 1.29–1.88), 1.52 in dizziness/balance disorder (95% CI, 1.27–1.82), 1.46 in rhinitis (95% CI, 1.28–1.65), and 1.82 in xerophthalmia symptoms (95% CI, 1.57–2.11).

**Table 3:** Association between ophthalmologic and otolaryngologic symptom experience and TMDs in Koreans aged ≥ 20 years participating in KNHANES V (2010–2012).

Factors			Univariate			Full model		
	Subgroup	N (TMD cases)	OR	95% CI		OR	95% CI	
Tinnitus	No (Ref.)	13,151 (1,349)	1			1		
	Yes	3,866 (654)	1.70	1.49	1.95	1.97	1.70	2.27
Hearing difficulty	No (Ref.)	14,456 (1,720)	1			1		
	Yes	2,605 (289)	0.90	0.75	1.07	1.55	1.29	1.87
Dizziness/balance disorder	No (Ref.)	9,512 (704)	1			1		
	Yes	2,613 (303)	1.40	1.17	1.68	1.52	1.27	1.82
Rhinitis symptoms	No (Ref.)	14,809 (1,284)	1			1		
	Yes	4,252 (725)	1.73	1.53	1.96	1.46	1.28	1.65
Xerophthalmia symptoms	No (Ref.)	13,209 (1,417)	1			1		
	Yes	2,872 (511)	1.82	1.57	2.12	1.82	1.57	2.12

Full models were adjusted for age, sex, personal income, education level, occupation, residence, marital status, BMI, alcohol intake, smoking status, and physical activity in estimation of odds ratios (ORs) and 95% confidence intervals (CIs).

Frequencies were analyzed for each variable using frequency analysis, and ORs and 95%Cis were calculated by complex sample design.

TMD, temporomandibular disorder; KNHANES V, 5th Korea National Health and Nutritional Examination Survey; OR, odds ratio; CI, confidence interval

### Association between TMD prevalence and abnormal findings on otolaryngological examination

Table 4 indicates the relationship between TMD prevalence and otolaryngological examination findings. The OR for TMD prevalence was 0.57 in subjects with normal laryngoscopic findings (95% CI, 0.36–0.90) and 1.44 in subjects diagnosed with chronic rhinosinusitis (95% CI, 1.11–1.87).

**Table 4:** Association between otolaryngological and dental examination and TMDs in Koreans aged ≥ 20 years participating in KNHANES V (2010–2012).

Factors	Subgroup		Univariate			Full model		
		N (TMD cases)	OR	95% CI		OR	95% CI	
Unilateral hearing loss	No (Ref.)	14,739 (1,800)	1			1		
	Yes	1,305 (117)	0.77	0.60	0.99	1.24	0.94	1.65
Pre-nasal astringent: pale edematous mucosa	No (Ref.)	14,476 (1,680)	1			1		
	Yes	2,479 (317)	0.98	0.80	1.20	0.87	0.70	1.07
Pre-nasal astringent: watery rhinorrhea	No (Ref.)	14,693 (1,688)	1			1		
	Yes	2,262 (309)	1.17	0.97	1.42	0.99	0.81	1.20
Pre-nasal astringent: mucoid or mucopurulent discharge	No (Ref.)	15,760 (1,848)	1			1		
	Yes	1,195 (149)	1.08	0.84	1.39	1.10	0.84	1.44
Abnormal laryngoscopy findings	No (Ref.)	10,126 (947)	1			1		
	Yes	722 (43)	0.52	0.34	0.80	0.57	0.36	0.90
Chronic rhinosinusitis	No (Ref.)	15,841 (1,830)	1			1		
	Yes	975 (146)	1.42	1.12	1.82	1.44	1.11	1.87
Nasal septum deviation	No (Ref.)	8,693 (1,033)	1			1		
	Yes	8,129 (943)	0.95	0.83	1.09	0.97	0.84	1.12
Permanent teeth caries	No (Ref.)	1,745 (160)	1			1		
	Yes	15,637 (1,873)	1.25	1.01	1.55	1.14	0.91	1.42
Periodontal disease	No (Ref.)	8,103 (1,295)	1			1		
	Yes	2,972 (318)	0.65	0.55	0.78	1.07	0.89	1.29

Full models were adjusted for age, sex, personal income, education level, occupation, residence, marital status, BMI, alcohol intake, smoking status, and physical activity in estimation of odds ratios (ORs) and 95% confidence intervals (CIs).

Frequencies were analyzed for each variable using frequency analysis, and ORs and 95%Cis were calculated by complex sample design.

TMD, temporomandibular disorder; KNHANES V, 5th Korea National Health and Nutritional Examination Survey; OR, odds ratio; CI, confidence interval

## Discussion

This is the first large-scale nationwide study to explore the relationship between chronic diseases, ophthalmologic and otolaryngological symptoms and examination findings, and TMD prevalence. Of the 17,575 survey respondents, 2,059 reported experience of ≥1 TMD symptom(s), and TMD prevalence was higher in patients with chronic diseases such as asthma, migraine, osteoarthritis, thyroid dysfunction, and depressive symptoms compared to non-patients. Furthermore, patients with ophthalmologic or otolaryngologic symptoms such as tinnitus, hearing difficulty, dizziness, and xerophthalmia, and those diagnosed with chronic rhinosinusitis upon otolaryngological examination exhibited higher prevalence of TMD, while patients with abnormal findings on laryngoscopy presented lower TMD prevalence.

### Asthma and TMDs

In the present study, TMD prevalence was shown to be higher in patients with asthma, which is contrary to a previous study on the relationship of temporomandibular joint osteoarthritis/osteoarthrosis with allergy and asthma by Nishioka et al. where allergy was identified as a risk factor, but no significant association was found for asthma [13]. Irritation of nerves around the temporomandibular joint may incur release of neuropeptides, which may in turn stimulate production and release of certain proinflammatory cytokines. Several cytokines including IL-1α, IL-6, IL-8, TNF-α, and IFN-γ were found to increase in the synovial fluid of patients with TMD, and these cytokines may be responsible for the synovitis and degenerative changes in cartilaginous tissue and bone of the temporomandibular joint. Similarly, as circulating lymphocytes from asthma patients also produce large amounts of interleukins, including IL-2, -4, and -5, the authors hypothesized that asthma may be a risk factor for temporomandibular joint osteoarthritis. However, perhaps due to the small sample size, the results did not reach statistical significance [13].

### Migraine and TMDs

The present study found TMD prevalence to be higher in subjects with migraine than those without, which is consistent with several previous study findings. Observations with regard to TMD tendencies in patients with tension headache or migraine due to temporalis pain have been made [17], and TMD treatment was found to decrease pain in some headache patients, further strengthening the relationship between headache and TMDs [18]. There are several possible explanations backing the relationship between migraine and TMD: Migraine and TMD pain are both mediated by the trigeminocervical nerve complex [19, 20], and the initially migraine-specific sensitization of trigeminal nerve nociception has been suggested to affect TMD pain also [21]. A 2015 study comparing MRI results of the mandibular joint in 20 TMD patients with and without migraine reported that overuse of the lateral pterygoid as a result of temporomandibular joint disc displacement could lead to migraine [22], while a 2012 study on twins revealed that only 12% of the genetic component of TMD pain was shared with migraine [23], suggesting a largely acquired, environmental association. Amongst psychological factors, depression and anxiety have been consistently identified as risk factors for migraine and TMD, and behavioral factors such as stress have also been reported to contribute to the pathogenesis of these conditions [24, 25]. The close relationship between migraine and TMDs appears to be grounded in various psychological and environmental elements and the anatomical proximity of involved nerves, and further serves as rationale for joint treatment of migraine with TMD.

### Osteoarthritis and TMDs

Various studies on the relationship between osteoarthritis and the temporomandibular joint have reported comparable positive correlations. A recent study on TMD symptoms in knee arthritis patients and non-arthritic controls reported that arthritic patients were more likely to experience temporomandibular joint dysfunction and limited range of motion [26], and joint space narrowing and osteophyte formation were observed in the temporomandibular joint in generalized osteoarthritis and rheumatoid arthritis patients [27]. However, osteoarthritic change of the temporomandibular joint and TMD symptoms do not always go hand in hand [28]. Prevalence of osseous changes resembling temporomandibular joint osteoarthritis was shown to be similar in both groups with and without TMD symptoms [29], indicating that radiological test results do not necessarily correlate with clinical symptoms. TMDs should therefore be identified as a syndrome, and managed with due consideration to its complexity and wide variety of symptoms as illustrated in the present study.

### Thyroid dysfunction and TMDs

While TMD prevalence was shown to be higher in patients with thyroid dysfunction than in those with normal thyroid function, there is a distinct paucity of studies looking into the potential relationship between thyroid dysfunction and TMD. Hypothyroidism is frequently accompanied by various musculoskeletal symptoms ranging from myalgia and joint pain to myopathy and osteoarthritis [30], and it can be carefully conjectured that TMDs may also occur as a musculoskeletal manifestation of thyroid dysfunction.

### Depressive symptoms and TMDs

Several previous studies have reported on the depressive tendencies of TMD patients compared to control groups [31–33]. Giannakopoulos et al. reported that females with chronic jaw pain were significantly more depressed than the general population, and under the same conditions, male patients tended to be more depressed than their female counterparts, implying that depressive symptoms are a stronger factor in determining chronic TMD pain than sex [34]. Yap et al. demonstrated that chronic TMD pain often coincides with psychological and psychosomatic disorders such as depression and somatization [35], which is in line with other TMD and orofacial pain studies where outcomes for concurrent psychological treatment and interdisciplinary treatments encompassing CBT, posture regulation, and biofeedback were superior to conventional dental care or usual care [14, 15].

### Tinnitus, hearing difficulty, and TMDs

The prevalence of TMD was higher in patients with tinnitus and hearing difficulty symptoms than in patients without tinnitus or hearing difficulty, which is consistent with previous TMD studies where reports of auditory complaints are common [36, 37]. Moreover, patients with TMDs were at greater risk of developing tinnitus and symptoms of greater severity than patients without TMDs [38, 39]. Excessive mechanical irritation of the discomallear ligament is suspected to play a definitive role in development of tinnitus in TMD patients [40, 41]. Tinnitus has also been linked with pressure and strain from mastication and jaw movement [42, 43]. In an audiological evaluation of aural symptoms in TMD, hearing loss was observed in up to 15–32% of TMD patients [44], and abnormal anatomy of the foramen tympanicum and ossification of the tympanic bone have been suggested to contribute to TMD occurrence [45, 46]. A previous study by Riga et al. attributed the hearing difficulty in TMD cases to increase in resonant frequency in the tympanum ipsilateral to the TMD [47]. The association between tinnitus, hearing difficulty and TMDs has thus been ascribed to bidirectional delivery of mechanical stimulation and stress due to anatomical proximity.

### Dizziness/balance disorder and TMDs

TMD prevalence was found to be higher in patients with dizziness/balance disorder than in those without. Otologic complaints most frequently cited with TMDs in the literature are dizziness, tinnitus, ear pain, ear fullness, and hearing loss [48, 49]. de Moraes Marchiori et al. purported that patients with TMDs are 2.38 times more likely to present with dizziness in a 2014 cross-sectional study [50], and Chole et al. conducted a case-control study to determine whether dizziness is more common in TMD patients compared to in age-matched controls, finding dizziness to be significantly more prevalent in TMD groups [51].

### Xerophthalmia and TMDs

TMDs were more prevalent in patients with xerophthalmia than in those without. Although there is no evidence directly supporting an association between xerophthalmia and TMD, Shigeishi described a relationship between TMDs and visual display terminal (VDT) syndrome which could serve as a connecting link between xerophthalmia and TMDs [52]. Other studies have suggested that prevalence of dry eye disease, and musculoskeletal and psychopathological symptoms is increasing in working individuals as a result of greater use of VDTs in work tasks such as programming, data input, and image construction. [53, 54]. TMD-related symptoms were observed in 16% of the VDT-using population [55].

### Laryngoscopy findings, chronic paranasal sinusitis, and TMDs

Surprisingly, TMDs were less prevalent in patients with abnormal laryngoscopy findings than in those with normal findings. The fact that laryngoscopic tests conducted by healthcare professionals begot a different conclusion from survey self-reports on otolaryngological disorders in association with TMDs is worth note, but may alternatively be due to small sample size. Patients with chronic rhinosinusitis had higher TMD prevalence than patients without chronic rhinosinusitis. Charleston et al. suggested that hypersensitivity to outside stimuli secondary to central nervous system sensitization may explain the pathophysiological interactions between myogenic TMDs and rhinosinusitis as both the orofacial musculature and paranasal sinuses are innervated by V1, V2 and the sphenopalatine ganglion [56]. Jeon et al. examined the relationship between TMDs and upper airway infection in 417 TMD patients and found that patients with limitations in opening the mouth were 9.93 times more likely to develop maxillary sinusitis/rhinitis than those without limitations (p = 0.0004) [57]. The authors explained the association as follows: as infection in the maxillary sinus may spread hematogenously or directly to the temporomandibular joint area and the pterygoid plate to which the external pterygoid muscle attaches is located close to the maxillary sinus, infection of the maxillary sinus may affect opening of the mouth based on the anatomical proximity of related structures.

### Strengths and limitations

The main strengths of this study are that it is the first survey to examine the association between TMDs and various diseases multidimensionally using data from a large-scale homogeneous sample representative of the South Korean population, and from a methodological standpoint, that KNHANES health examinations and surveys were systematically implemented by trained professionals. This study included otolaryngological examination findings in addition to self-reported data in a national-level survey. The authors would also like to draw attention to the fact that the following procedures were installed to the aim of heightening examiner reliability: public health dentists who participated as dental examiners received biannual education and training sessions specifically for the present study with a final expectation level of kappa coefficient≥0.85 with the standard examiner, and otolaryngology residents who performed the otolaryngological examinations underwent annual training sessions. In addition, self-report of TMD pain displayed high reliability and validity with a sensitivity of 0.98 and specificity of 0.90 compared to the reference standard of the RDC/TMD, which has been demonstrated to be valid for common pain-related and intra-articular TMDs, although evidence is currently limited to adolescents [58]. Further, tension-type headaches are a common reason for false-positive results based on self-report TMD pain questions [59], and acts as a limitation of this study. Tension-type headache has a global prevalence of 42%, but estimates differ widely across the world [60]. Results from the PACE study indicate a tension-type headache prevalence of 19.4% at the lower data range limit for adults in Western countries, and estimates for headaches in children and adolescents have been reported to vary considerably by age and setting [61]. Still, various confounding factors, such as age, sex, income, education level, occupation, residence, marital status, BMI, alcohol consumption, smoking status, and physical activity, were additionally adjusted for in analysis.

However, the present study also holds various limitations, including its cross-sectional design where a random time period is assessed rendering it difficult to infer causality and only enabling study of associations. The biggest limitation of this study may be the discrepancy in diagnostic criteria between this study and that for the widely used and well-recognized classification of RDC/TMD, as the study would have greatly benefited from further subgroup analyses by major RDC/TMD groups such as myofascial and disc displacement groups. Especially as patients with myogenous TMD reported significantly more comorbid disorders in neurologic, gastrointestinal, musculoskeletal, and psychologic categories, and more severe pain compared to arthrogenous TMD patients [62]. However, subgroup analysis of the myofascial group and disc displacement group could not be performed due to inherent study limitations from retrospective use of secondary national healthcare data where TMD data was collected by means of self-reported surveys not consistent with RDC/TMD [63], and readers should take the fact that the TMDs assessed in this study were not as structurally definitive into due consideration in interpretation of these results. On a different note, blood pressure measurement standards were somewhat inconsistent as difference in blood pressure depending on the height of the heart and arm was not adjusted for in 2010 as in the 2011–2012 measurements. Such limitations are inherent and shared with other studies employing secondary data extracted from existing surveys. An additional limitation is that chronic disease prevalence was determined from survey items as opposed to medical diagnosis.

Despite these limitations, the authors hope that these findings may be of use as reference to clinicians and researchers as they contain otolaryngological and dental examination results collected from a large-scale, nationally representative population, and this study may be the grounds for revision of the TMD section of KNHANES towards incorporating more concurrent terminology and definitions in assessment of TMDs in large surveys for wider clinical relevance and implications. Potential mechanisms and pathways underlying the relationship between various chronic and ophthalmologic and otolaryngological disorders and TMDs should be further investigated to determine the causal relationship between TMDs and other diseases by means of prospective clinical studies.

## Conclusion

This study assessed the association between TMD prevalence and various disorders. Chronic diseases and ophthalmologic and otolaryngologic symptoms were associated with higher TMD prevalence with the exception of otolaryngological examination findings. These findings suggest that TMD treatment should employ an interdisciplinary approach in an effort to extend and maximize its effect.
